# Phytocompounds from Himalayan Medicinal Plants as Potential Drugs to Treat Multidrug-Resistant *Salmonella* *typhimurium*: An In Silico Approach

**DOI:** 10.3390/biomedicines9101402

**Published:** 2021-10-05

**Authors:** Jyoti Mehta, Rajan Rolta, Deeksha Salaria, Oladoja Awofisayo, Olatomide A. Fadare, Prem Prakash Sharma, Brijesh Rathi, Adity Chopra, Neha Kaushik, Eun Ha Choi, Nagendra Kumar Kaushik

**Affiliations:** 1 Faculty of Applied Sciences and Biotechnology, Shoolini University, Himachal Pradesh 173212, India; yjjyoti@gmail.com (J.M.); roltarajan612@gmail.com (R.R.); deekshasalaria20@gmail.com (D.S.); 2 Department of Pharmaceutical and Medical Chemistry, University of Uyo, Uyo 520003, Nigeria; oladojaawofisayo@uniuyo.edu.ng; 3 Organic Chemistry Research Lab, Department of Chemistry, Obafemi Awolowo University, Osun 220282, Nigeria; tofadare@oauife.edu.ng; 4 Laboratory for Translational Chemistry and Drug Discovery, Hansraj College, University of Delhi, Delhi 110007, India; premprakash.cr@gmail.com (P.P.S.); brijeshrathi@hrc.du.ac.in (B.R.); 5 Laboratory of Computational Modelling of Drugs, South Ural State University, 454080 Chelyabinsk, Russia; 6 Department of Immunology, University of Oslo, 0315 Oslo, Norway; aditychopra.chopra@gmail.com; 7 Department of Biotechnology, College of Engineering, Suwon University, Hwaseong-si 18323, Korea; neha.bioplasma@suwon.ac.kr; 8 Plasma Bioscience Research Center & Applied Plasma Medicine Center, Department of Electrical and Biological Physics, Kwangwoon University, Seoul 01897, Korea; ehchoi@kw.ac.kr

**Keywords:** multidrug resistance, efflux pump, medicinal plants, phytocompounds, molecular docking, drug likeness, toxicity, MD simulation

## Abstract

Medicinal plants can be used as natural therapeutics to treat diseases in humans. Enteric bacteria possess efflux pumps to remove bile salts from cells to avoid potential membrane damage. Resistance to bile and antibiotics is associated with the survival of *Salmonella enterica* subspecies *enterica* serovar Typhimurium (*S. typhimurium*) within a host. The present study aimed to investigate the binding affinity of major phytocompounds derived from 35 medicinal plants of the North Western Himalayas with the RamR protein (PDB ID 6IE9) of *S. typhimurium*. Proteins and ligands were prepared using AutoDock software 1.5.6. Molecular docking was performed using AutoDock Vina and MD simulation was performed at 100 ns. Drug likeness and toxicity predictions of hit phytocompounds were evaluated using molinspiration and ProTox II online servers. Moreover, docking, drug likeness, and toxicity results revealed that among all the selected phytocompounds, beta-sitosterol exhibited the most efficacious binding affinity with RamR protein (PDB ID 6IE9) and was nontoxic in nature. MD simulation data revealed that beta-sitosterol in complex with 6IE9 can be used as an antimicrobial. Furthermore, beta-sitosterol is stable in the binding pocket of the target protein; hence, it can be further explored as a drug to inhibit resistance-nodulation-division efflux pumps.

## 1. Introduction 

*Salmonella* is a bacterial pathogen that infects the intestinal tract and gallbladder and causes numerous foodborne illnesses in humans. Enteric bacteria, such as *Salmonella*, tolerate the existence of bile acids for their survival in the gastrointestinal transit and gallbladder [1,2]. Nontyphoidal serovars (NTSs) of *Salmonella enterica* are the major causes of foodborne illnesses and diarrhea occurring worldwide [3,4]. In *S. enterica*, the resistance-nodulation-division (RND) pump is translated from the acrAB gene regulated by RamA, a transcriptional activator. RamR inhibits the expression of the ramA gene involved in multidrug resistance in *Salmonella enterica* subspecies *enterica* serovar Typhimurium (*S. typhimurium*). *S. typhimurium* is one of the NTSs causing severe human infections and results in more hospitalizations and mortality worldwide [5]. Additionally, the treatment choices are limited because antibiotics may lead to enhanced shedding of *S. typhimurium* and its emergence as multidrug-resistant bacteria [6,7]. Presently, it is no longer considered as the first choice of antimicrobial drug due to its resistance. The advent of new resistance mechanisms exists in *S. typhimurium*, leading to challenges in treating infections. Therefore, alternative therapeutic approaches are required. *S. typhimurium* comprises of at least nine multidrug efflux systems [8]; among these, the AcrAB-TolC system, containing the AcrB transporter of the RND family, is particularly effective in developing resistance to bile acid [8,9]. RamR is a local transcriptional repressor, which belongs to the TetR family of regulatory proteins [10]; it helps in impairing the ramA gene expression that affects ramA gene transcription resulting in multidrug resistance. Therefore, it is crucial to identify novel pharmacological targets against drug-resistant *S. typhimurium*.

Medicinal plants have played a pivotal role in treating diseases since the prehistoric period. These plants comprise various phytoconstituents in every part (bark, leaves, flowers, roots, fruits, and seeds), and exhibit high therapeutic value [11].

Herbal medications have recently gained immense interest as they are safe and economic and have been widely used for several years to treat diseases. Bioactive components are secondary metabolites of plants that produce pharmacological and toxicological issues in living organisms. It is difficult to screen each phytoconstituent for toxicity. In drug design, computational techniques play a crucial role in studying the toxicity of chemical and natural compounds as well as their properties [12]. In silico studies, with specific reference to toxicity prediction and molecular docking for each phytochemical in order to determine their therapeutic efficiency, require less time, are economic, and can harm animals [13,14]. Therefore, the present study was designed to investigate the binding affinity of phytocompounds of 35 important medicinal plants of the Northwestern Himalaya with *S. typhimurium* protein (RamR transcriptional repressor of TetR family) with PDB ID: 6IE9 to prevent inflammatory gastroenteritis. 

## 2. Methodology

### 2.1. Bioinformatics Tools

Open Babel GUI [15], UCSF Chimera 1.8.1, Pubchem (www.pubchem.com (accessed on 15 May 2021), RCSB PDB (http://www.rscb.org/pdb (accessed on 15 May 2021)), Autodock/vina software [16], and Discovery Studio were used in the present investigation.

### 2.2. Ligand Preparation

Seventy major phytocompounds of 35 medicinal plants from Himachal Pradesh, India, were selected for molecular docking analysis. The three-dimensional structures of all the phytocompounds and resistant bile components of *S. typhimurium* (chenodeoxycholic acid) were downloaded from Pubchem (www.pubchem.com (accessed on 15 May 2021)) in .sdf format, which was finally converted into a PDB file. Each selected ligand (phytocompounds and bile component) was prepared using the open Babel software from the command line on an Ubuntu terminal. Table 1 lists the names of the phytocompounds selected for this study, their plant sources, pharmacological properties, and ethnomedicinal uses.

### 2.3. Protein Preparation

RamR of *S. typhimurium* [67] was used for molecular docking with major phytocompounds from 35 important medicinal plants (Table 1) found in the northwestern Himalayas of Himachal Pradesh to identify potential inhibitors of *S. typhimurium.* The 3-D structure protein (PDB ID = 6IE9) was downloaded from the protein databank (http://www.rscb.org/pdb (accessed on 15 May 2021)) as a pentamer, and chain A was extracted for docking using PyMol. Chain A was prepared for docking, and a grid box was set to cover the entire protein (grid box dimensions = 40, 40, 40 Å) and was centered at x, y, z = 11.029, 33.324, 12.359 Å, respectively.

### 2.4. Molecular Docking of Major Phytocompounds of Thirty-Five Medicinal Plants

The AutoDock tool was used to dock the selected ligands to the catalytic triad of proteins, which was further stored as a pdbqt file. Docking was carried out to estimate the population of possible ligand conformations/orientations at the binding site. To align the ligands in the same spatial coordinates, a vina perl script was used [16]. The best conformation was selected with the minimum docked energy after completing the docking search. The pdb complex of protein and ligands was analyzed using Discovery Studio (https://discover.3ds.com/d (accessed on 15 May 2021)) to study the interactions between proteins and ligands. The binding strength of the ligand was calculated as a negative score (kcal/mol).

### 2.5. Drug Likeness Calculations

The drugs were scanned to assess whether the selected phytochemicals met the drug-likeness criteria. Lipinski’s rule of 5 using Molinspiration (http://www.molinspiration.com (accessed on 15 May 2021)) was used to verify drug likeness attributes, such as the number of hydrogen acceptors <10, number of hydrogen donors <5, molecular weight <500 Da, and partition coefficient log P > 5. The smiles format of all major phytocompounds was uploaded for further screening [68]. 

### 2.6. ADMET Screening and Toxicity Prediction of Phytocompounds

**Figure 1:**
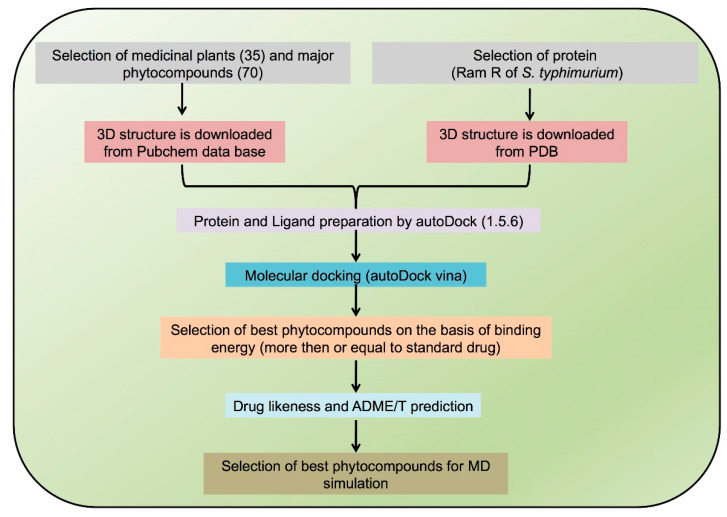
Schematic of experimentation.

Absorption, distribution, metabolism, excretion, and toxicity (ADMET) screening was performed to evaluate the absorption, toxicity, and drug-likeness properties of the selected phytocompounds. The 3-D structures of 11 phytocompounds (asiaticoside, beta-sitosterol, bryophyllin A, madecassoside, Mahanimbine, Pennogenin, Rutin, Solasonine, Solamargine, Withaferin A, and Withanone) were saved in smiles format and uploaded on the SWISSADME (http://www.swissadme.ch/ (accessed on 15 May 2021)) (Molecular Modeling Group of the SIB (Swiss Institute of Bioinformatics) and PROTOX-II (https://tox-new.charite.de/protox_II/) web servers (Charite University of Medicine, Institute for Physiology, Structural Bioinformatics Group, Berlin, Germany) [69,70,71,72] for ADMET screening. SWISSADME is an online tool used to predict ADME and pharmacokinetic and physicochemical features of a molecule, which are the main determinants for clinical trials. Toxicity was evaluated in compounds with LD_50_ values ≤50 mg/kg (Class I), >50 mg/kg but <500 mg/kg (Class II), 500 < LD_50_ ≤ 5000 mg/kg (Class III), and LD_50_ > 5000 mg/kg (Class IV). Classes I, II, and III exhibited less toxicity, whereas Class IV revealed no toxicity [73,74]. Moreover, PROTOX is a rodent oral toxicity server that determines the LD_50_ value and the toxicity class of a target molecule [69]. A schematic of the experiment is illustrated in Figure 1.

### 2.7. MD Simulation of Protein Ligand Complexes 

The ligand–protein complex structure was prepared before MD simulation to remove the structural errors. Extensive 100 ns MD simulation was performed on the Desmond platform to analyze the ligand behavior within the complex [75]. The complex was solvated in a TIP3P (8018 molecules) water model and 0.15 M NaCl (Na: 54.42 mMol and Cl: 49.88 mMol) to mimic a physiological ionic concentration. The molecular mechanics/generalized born surface area (MM/GBSA) was used for binding free energy calculations [75]. MD simulation trajectories were used as inputs to calculate the MM/GBSA of the binding free energies of the ligands and to investigate their binding mechanisms. The thermodynamic binding energy was calculated for every 1000th frame, as the complete MD simulation includes 10,000 frames. The OPLS 2005 force field was used for the MD simulations.

## 3. Results

### 3.1. Molecular Docking of 70 Major Phytocompounds from 35 Medicinal Plants with Ram R Protein of S. typhimurium

**Figure 2:**
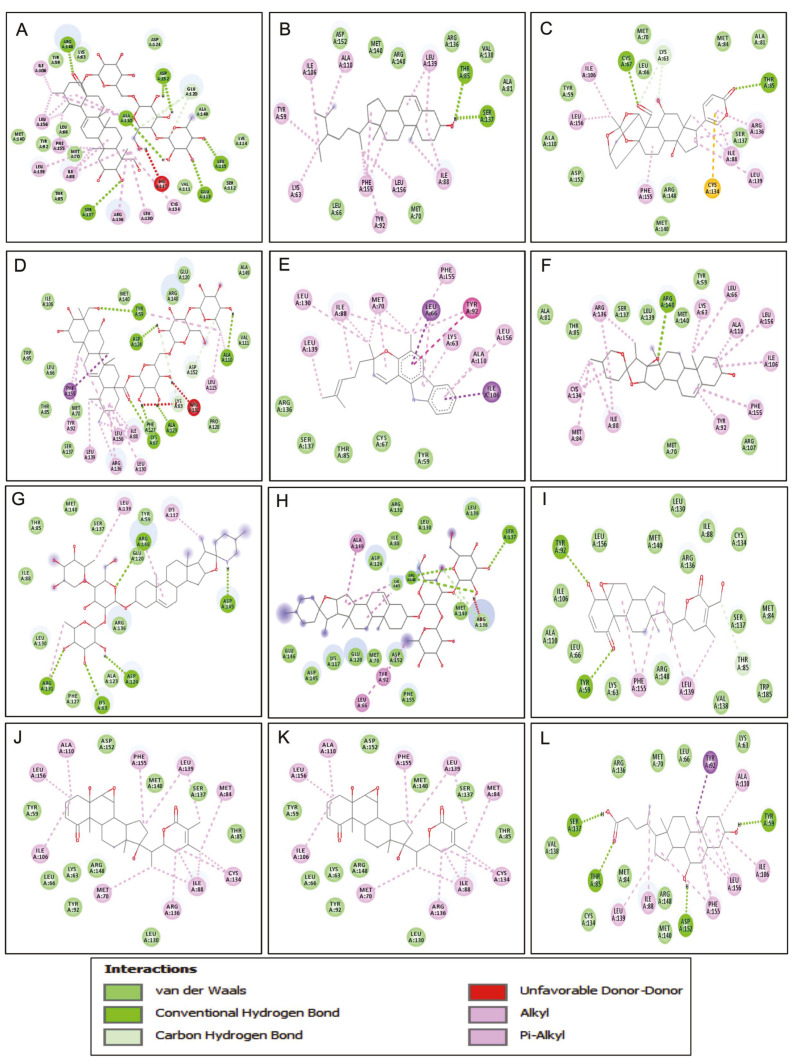
Two-dimensional structure of 11 phytocompounds and bile components in complex with RamR protein of *Salmonella* Typhimurium: (**A**) asiaticoside, (**B**) beta-sitosterol, (**C**) bryophyllin A, (**D**) madecassoside, (**E**) mahanimbine, (**F**) pennogenin, (**G**) rutin, (**H**) solamargine, (**I**) solasonine, (**J**) withaferin A, (**K**) withanone, and (**L**) chenodeoxycholic acid.

Molecular docking was performed with the RamR protein of *S. typhimurium* and AutoDock vina software to study the interactions of the major phytocompounds of 35 medicinal plants of the northwestern Himalayas in efflux pump inhibition. The docking results revealed that out of 70 phytocompounds from 35 medicinal plants, only 11 phytocompounds from 8 medicinal plants revealed binding energy comparable to that of the bile component chenodeoxycholic acid. Asiaticoside from *Centella asiatica* exhibited the highest binding energy (−10.9 KJ/mol), followed by the bile component chenodeoxycholic acid (−10.8 KJ/mol), bryophyllin A (−10.6 KJ/mol), pennogenin (−10.3 KJ/mol), withaferin A (−10.2 KJ/mol), madecassoside and solasonine (−9.7 KJ/mol), solamargine (−9.5 KJ/mol), mahanimbine (−9.4 KJ/mol), withanone (−9.3 KJ/mol), rutin (−9.2 KJ/mol), and beta-sitosterol (−9.2 KJ/mol). Interactive amino acids are listed in Table 2 and Figure 2.

**Figure 3:**
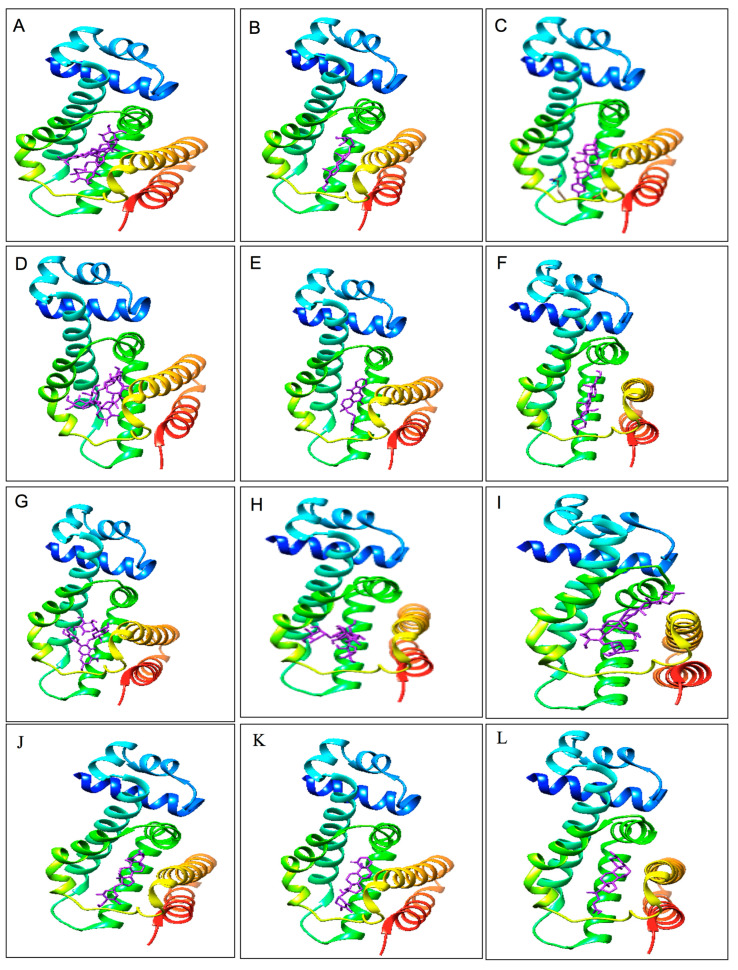
Ribbon structure of 11 active phytocompounds and bile component in complex with RamR protein of *Salmonella* Typhimurium: (**A**) asiaticoside, (**B**) beta-sitosterol, (**C**) bryophyllin A, (**D**) madecassoside, (**E**) mahanimbine, (**F**) pennogenin, (**G**) rutin, (**H**) solamargine, (**I**) solasonine, (**J**) withaferin A, (**K**) withanone, and (**L**) chenodeoxycholic acid.

**Figure 4:**
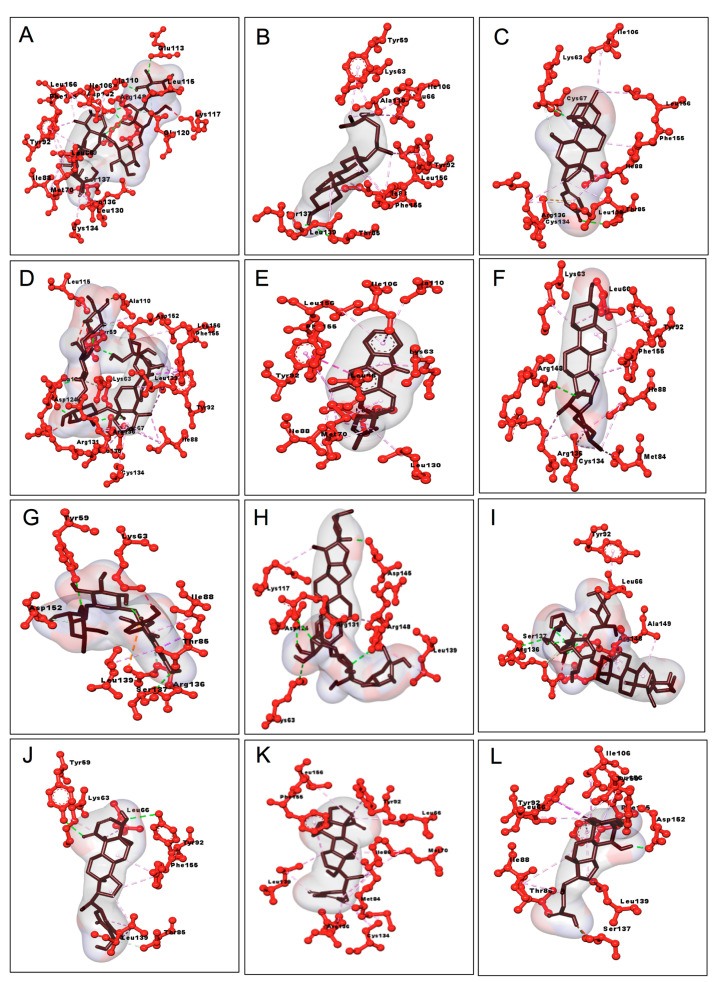
Three-dimensional interactions of active phytocompounds and standard drugs in complex with RamR protein of *Salmonella* Typhimurium: (**A**) asiaticoside, (**B**) beta-sitosterol, (**C**) bryophyllin A, (**D**) madecassoside, (**E**) mahanimbine, (**F**) pennogenin, (**G**) rutin, (**H**) solamargine, (**I**) solasonine, (**J**) withaferin A, (**K**) withanone, and (**L**) chenodeoxycholic acid.

Asiaticoside in complex with 6IE9 revealed hydrogen bonding with Ser137, Glu113, Leu115, Asp152, Ala110, and Arg148 and hydrophobic interactions with Ile106, Tyr59, Lys63, Asp124, Glu120, Ala149, Lys114, Ser112, Val111, Lys117, Cys134, Leu130, Arg136, Thr85, Ile88, Met70, Leu139, Phe155, Leu66, Tyr92, Leu156, and Met140. Similarly, other phytocompounds, such as madecassoside, exhibited hydrogen bonding with Tyr59, Asp124, Ala110, Ala123, and Cys67 and hydrophobic interactions with Trp95, Leu66, Phe155, Thr85, Met70, Tyr92, Ser137, Leu139, Arg136, Leu130, Leu156, Ile88, Phe127, Lys63, Arg131, Pro128, Leu115, Asp152, Val11, Ala149, Glu120, Arg148, Met140, and Ile106. Beta-sitosterol revealed hydrogen bonding with Thr85 and Ser137 and hydrophobic interactions with Val138, Ala81, Ile88, Leu156, Met70, Tyr92, Phe155, Leu66, and Lys63. Bryophyllin A exhibited hydrogen bonding with Thr85 and Cys67 and hydrophobic interactions with Phe155, Arg148, Met140, Cys134, Ile88, Leu139, Ser137, Arg136, Ala81, Met84, Lys63, Leu66, Met70, Ile106, Tyr59, Leu156, Ala110, and Asp152. Moreover, mahanimbine revealed hydrophobic interactions with Arg136, Ser137, Thr85, Cys67, Tyr59, Ile106, Ala110, Leu156, Lys63, Tyr92, Leu66, Phe155, Met70, Ile88, Leu139, and Leu130. Pennogenin exhibit hydrogen bonding with Arg148 and hydrophobic interactions with Cys134, Met84, Ile88, Met70, Tyr92, Arg107, Phe155, Ile106, Leu156, Ala110, Leu66, Lys63, Met140, Tyr59, Leu139, Ser137, Arg136, Thr85, and Ala81. Rutin presented hydrogen bonding with Ser137, Thr85, and Tyr59 and hydrophobic interactions with Cys134, Met84, Glu120, Asp152, Arg148, Ala110, Leu156, Ala123, Asp124, Tyr92, Leu66, Phe155, Met70, Lys63, Cys67, Phe127, Arg136, Leu130, Ile88, and Leu139. Furthermore, solasonine revealed hydrogen bonding with Arg148 and Ser137 and hydrophobic interaction with Phe127, Ala123, Leu130, Arg136, Glu120, Lys117, Tyr59, Leu139, Ser137, Met140, Thr85, and Ile88. Solamargine exhibited hydrogen bonding with Arg131, Lys63, Asp124, Asp145, and Arg148 and hydrophobic interactions with Glu146, Asp145, Lys117, Glu120, Met70, Tyr92, Leu66, Phe155, Asp152, Met140, Arg136, Leu139, Leu130, Lys68, Ile88, Arg131, Asp124, and Ala149. Withaferin A presented hydrogen bonding with Tyr59 and Tyr92 and hydrophobic interactions with Ile106, Ala110, Leu66, Lys63, Phe155, Arg148, Leu139, Val138, Thr85, Trp185, Ser137, Met84, Cys134, Arg136, Ile88, Leu130, Met140, and Leu156. Withanone exhibited hydrophobic interactions with Ile106, Leu66, Lys63, Arg148, Tyr92, Met70, Leu130, Arg136, Ile88, Cys134, Thr85, Met84, Ser137, Leu139, Met140, Phe155, Asp152, Ala110, Leu156, and Tyr59. Chenodeoxycholic acid revealed hydrogen bonding with Ser137, Thr85, Asp152, and Tyr59 and hydrophobic interaction with Val138, Cys134, Met84, Leu139, Ile88, Arg148, Met140, Phe155, Leu156, Ile106, Ala110, Lys63, Tyr92, Leu66, Met70, and Arg136. The ribbon and 3-dimensional structure of 11 major phytocompounds and bile components in complex with 6IE9 are illustrated in Figure 3 and Figure 4. Furthermore, all the selected phytocompounds were screened for drug likeness and ADME/T.

### 3.2. Drug Likeness Prediction of Active Phytocompounds of Eight Medicinal Plants

Drug likeness was predicted using Molinspiration (server) to study the drug-likeness properties of active phytocompounds, which are based on the Lipinski rule of 5. Lipinski’s rule of five was followed by all selected phytocompounds and the bile component, chenodeoxycholic acid, which revealed no violation. Among all the phytocompounds, bryophyllin A, pennogenin, withaferin A, and withanone followed all the rules of drug likeness, whereas beta-sitosterol and mahanimbine exhibited one violation, which was acceptable (Table 3).

### 3.3. Toxicity Prediction of Active Phytocompound and Chenodeoxycholic Acid

The toxicity of phytocompounds was predicted using ProTox-II server and the results are summarized in Table 4. Among all the phytocompounds, only beta-sitosterol exhibited one violation and immune toxicity. Based on the molecular drug likeness and toxicity data, beta-sitosterol was found to be the best phytocompound, which can be used for efflux pump inhibition, and it was further selected for molecular dynamics studies. 

### 3.4. MD Simulation of Protein–Ligand Complexes

**Figure 5:**
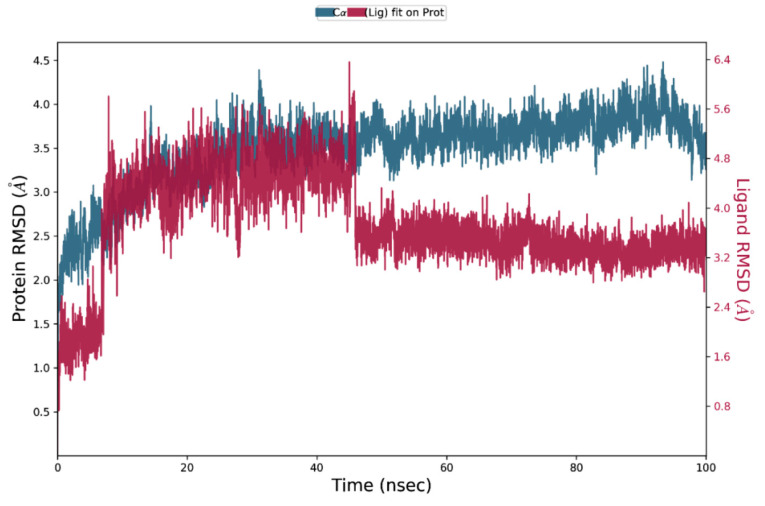
Root-mean-square deviation plot of C-alpha of beta-sitasterol in complex with 6IE9at 100 ns depicting the quality of the pose with respect to time.

**Figure 6:**
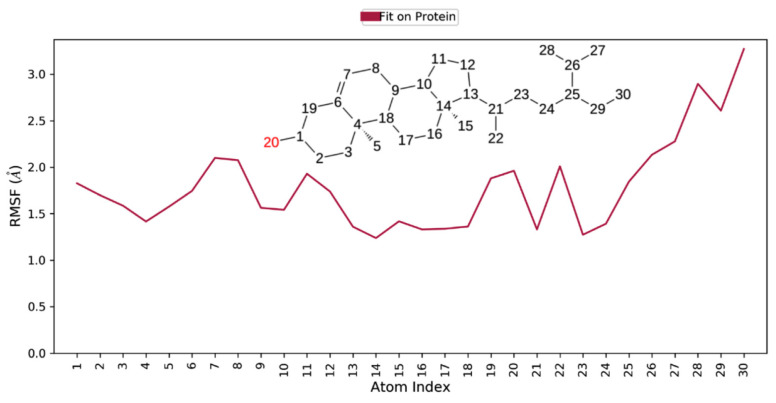
Ligand RMSF plot of beta-sitasterol in complex with 6IE9: *X*-axis depicts atom index and *Y*-axis reveals RMSF (Å).20 stands for atom number which is oxygen.

**Figure 7:**
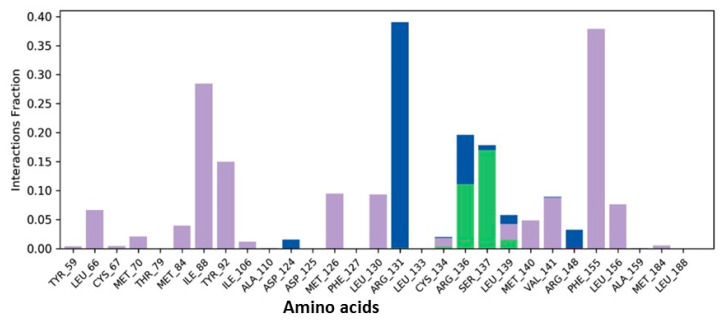
Histogram plot of beta-sitasterol in complex with 6IE9: *X*-axis depicts interactive amino acids and *Y*-axis reveals interaction fractions. (Gray color indicates Hydrophobic interactions, Blue color indicates water bridges and Green color indicates Hydrogen bonding).

Molecular dynamics simulation provides insight into the protein–ligand stability and protein structural flexibility of the docked complexes. The root-mean-square deviation (RMSD) plot of beta-sitasterol and 6IE9 complex exhibited significant stability in the protein pocket. These compounds fluctuate within the acceptable range between 3.2 and 5.6 Å, whereas protein Cα RMSD became stable after 25 ns and fluctuates in the range between 4.8 and 6.0 Å (Figure 5). The trajectory analysis revealed that a sharp change in the ligand RMSD at approximately 45 ns mainly occurred due to the aliphatic chain; this was also observed in the ligand RMSF plot, where fluctuation occurs in atoms 26–30 (Figure 5 and Figure 6). Several interactions were responsible for the conformational stability of the compound within the binding pocket, where hydrophobic interactions developed with residues L66, I88, Y92, M126, L130, Val141, Phe155, and L156 (Figure 7). The hydroxyl group revealed hydrogen bonding and water interactions with residues R136, S137, and R131. Moreover, MD simulation supported the docking results, where the compound interacted with residues that were linked with the molecule (Figure 2B and Figure 7). Furthermore, the thermodynamic energy analysis revealed that the average binding free energy was 138.65 ± 19.84 kcal/mol, whereas that of the docked complex was 109.18 kcal/mol (Table 5).

## 4. Discussion

Poor pharmacological characteristics are the major cause of late-stage failure in drug discovery. Thus, early determination of the inherent medicinal activities of the target compounds is crucial [76]. Moreover, medicinal plant species are abundant in Asia’s Himalayan woodlands, and they play a pivotal role in rural livelihoods by producing various valuable food and pharmaceutical commodities [77]. In recent years, the WHO estimated a remarkable increase in the multidrug resistance rate worldwide due to *Salmonella* strains [78]. *Salmonella* infections are gaining importance worldwide owing to their socioeconomic impact. *Salmonella* Typhimurium is one of the most common serovars predominantly associated with clinically reported human salmonellosis in several countries, accounting for at least 15% of infections worldwide [5]. Moreover, at least nine multidrug efflux pumps confer drug resistance in *Salmonella*; among these, AcrAB is constitutively expressed and is the most potent drug for intrinsic drug resistance [8]. AcrAB is a member of the RND family transporter that cooperates with TolC, an outer membrane component [79]. The AcrAB-TolC system comprising RND transporters can accumulate substrates in the periplasm rather than in the membrane or cytoplasm [80]. A common mechanism of intrinsic resistance to antimicrobial agents in Gram-negative bacteria is represented by the RND family efflux systems, which extrude a broad spectrum of antibiotics and biocides from the periplasm to the exterior of the cell [81].

The present study explored some medicinal plants, including *Girardinia diversifolia,* which we reported earlier for synergistic and efflux pump inhibitory activity against different strains of *S. typhimurium* and *Staphylococcus aureus* [82,83]. In contrast to our study, Mehta et al. [84] reported that methanolic extracts of *Pistacia integerrima*, *Ocimum sanctum*, *C. asiatica*, *Momordica charantia*, *Zingiber officinale*, and *Withania somnifera* exhibited synergistic activity in combination with ciprofloxacin and tetracycline against multidrug resistance. AcrAB-TolC in *Salmonella* Typhimurium acts as an efflux pump inhibitor. Furthermore, they reported the binding affinity (−8.2 kcal mol^−1^) of lariciresinol with 6EI9 (RamR). Similarly, Luhata et al. [85] reported the antibacterial activity of beta-sitosterol against *S. aureus*. Sen et al. [86] reported the antibacterial activity of beta-sitosterol against *Escherichia coli*, *Pseudomonas aeruginosa*, *S. aureus*, and *Klebsiella pneumoniae*. Rolta et al. [87] studied the antibacterial and antifungal activities of phytocompounds of *Rheum emodin* (emodin, rhein-13c6, and chrysophenodimethy ether) by molecular docking and MD simulations and found that phytocompounds of *R. emodin* exhibited the best interaction with bacterial and fungal targets. Similarly, Rolta et al. [71] studied the interactions of phytocompounds with the N-protein of severe acute respiratory syndrome coronavirus 2 (SARS-CoV-2) 2020, and reported that emodin, aloe-emodin, anthrarufin, alizarine, and dantron phytocompounds efficiently inhibit SARS-CoV-2 N-protein. Salaria et al. [88] studied the in vitro and in silico antibacterial and antifungal activities of essential oil, thymol derived from *Thymus serpyllum*, and validated the docking results via MD simulations. The conformational changes during protein–ligand interactions have been extensively studied via MD simulation methods [89].

## 5. Conclusions

The major phytocompounds of 30 fine medicinal plants of the northwestern Himalayas were selected for molecular docking study with the 6EI9 (RamR) target protein of *S. typhimurium*. Among all the selected phytocompounds, 11 phytocompounds exhibited the best activity compared to the standard drugs. Drug likeness and toxicity data revealed that beta-sitosterol, a major phytocompound of *G. diversifolia* (Link) Friis, is nontoxic in nature and follows the drug likeness rule. Moreover, MD simulation of beta-sitosterol in complex with 6EI9 was found to be stable between 0 and 100 ns time period. In this study, we found that beta-sitosterol is a potential plant-based drug for treating *S. typhimurium* infection. Furthermore, this study needs to be validated through in vitro and in vivo experiments.

## Figures and Tables

**Table 1:** Medicinal plants used for molecular docking with their uses in various ailments.

S. No.	Phytocompounds	Botanical Names (Family)	Common/Local Name	Ailments
1.	(Z)-Ligustilide	*Angelica glauca* Edgew. (Apiaceae)	Chora	Stimulant, appetizer, arthritis, carminative, diaphoretic, diuretic, constipation, debility, joint problems, bronchitis, dysentery, menorrhea, stomach disorders, vomiting [17,18].
	Angelicide			
2.	P-coumaric acid	*Heracleum lanatum* Michx (Apiaceae)	Patrala	Fever, abdominal cramps, leukoderma, aphrodisiac, digestive, mildly expectorant and sedative, nausea, tumor [19].
	Scopoletin			
3.	Palmatine	*Berberis aristata DC.* (*Berberidaceae*)	Kashmal	Acidity, eye infection, microbes, fever, hepatotoxic, hyperglycemic, lipidemic, cancer, oxidative stress diarrhea, hemorrhoids, osteoporosis, HIV-AIDS, diabetes, jaundice, wound healing [20].
	Rutin			
4.	Podophyllotoxin	*Sinopodophyllum hexandrum* (Royle) T.S. Ying (*Berberidaceae*)	Bankakdi	Cancer, snakebite, jaundice, stomachache, intestinal purgative, vomiting, necrotic wounds, tumor, arthritis [21,22].
	Quercetin			
5.	Astragalin	*Chenopodium album* L. (*Amaranthaceae*)	Baathu	Parasitic worms, inflammation, fever, arthritis, constipation, toothache, bug bites, sunstroke, tooth decay [21].
	Kaempferol			
6.	Apigenin	*Sedum glaucophyllum* R.T. Clausen (Crassulaceae)	Mochu-gha, ludru	Burn, cut, abscesses, blisters [23].
	Luteolin			
7.	Phytol	*Solena amplexicaulis* (Lam.) Gandhi (Cucurbitaceae)	Kakdi	Cancer, oxidative stress, inflammation, tumor, antimicrobial, diuretic, fever, jaundice [24].
	Carane			
8.	Hydroxytyrosol	*Malva neglecta* Wall (Malvaceae)	Sonchal	Constipation, women sterility, wound healing, hemorrhoids, asthma, diarrhea, rheumatic pain, stomachache, abdominal pain, renal diseases, throat infection, common cold, stomachache, antimicrobial, oxidative stress, inflammation, stress, liver damage, ulcer, cancer, bronchitis, kidney stone [25,26].
	Hexatriacontane			
9.	Cyanidin	*Ficus carica* L. (Moraceae)	Common fig	Oxidative stress, Cancer, colic, indigestion, loss of appetite, diarrhea, sore throats, coughs, bronchial problems, heart disease, liver problem, lowering of blood sugar, cholesterol-lowering, inflammation, Antimicrobial, relieve spasm of involuntary muscle, fever, TB, platelet aggregation inhibitor, mutagen [27,28].
	Psoralen			
10.	Militarine	*Dactylorhiza hatagirea* (D.Don) Soo (Orchidaceae)	Panja, Salampanja	Wound healing, inflammation, bleeding, fever, cancer, diabetes, neurological function, burns, and bronchitis [29,30].
	Resveratrol			
11.	Gallic acid	*Rheum australe* D.Don (Polygonaceae)	Chuchi, Chukari	Diabetes, inflammation, oxidative stress, cancer, gastric disorder, cuts & wounds, fractured bones, liver damage, immune-enhancing, lower blood glucose, smallpox, muscle sprain [31,32].
	Rhein			
12.	Nepodin	*Rumex hastatus* D.Don (Polygonaceae)	Almoru	Jaundice, hepatitis, Blood purification, Scurvy, Diuretic, cooling, astringent, constipation, oxidative stress, snakebites, foot and mouth infections, asthma, cough, headache, diarrhea, dysentery, fever, weakness, and scabies [33].
	Rumexoside			
13.	Kutkoside	*Picrorrhizakurooa* Royle (Plantaginaceae)	Karu	Liver damage, oxidative stress, cancer, asthma, stimulate immune system, neuritogenic, neuron degeneration, jaundice, allergy, piles, leukoderma, snake bite, liver disease, fever, parasitic worms, improving heart muscle contraction, high blood pressure, diabetes, cold, cough, stomach ache [34].
	Picroside III			
14.	Aloesin	*Rumex nepalensis* Meisn (Polygonaceae)	Nepal dock	Purgative, oxidative stress, fever, inflammation, tumor, diabetic, mental disorder, Wound healing, analgesic and CNS depressant, skeletal muscle relaxant [35].
	Orcinol glucoside			
15.	Catechin	*Rubus ellipticus* Sm. (Rosaceae)	Akhe, Yellow Himalayan raspberry	Dysentery, oxidative stress, diabetes, tumor, Nephroprotective, sore throats, cold, colic, constipation, gastritis, dysentery, diarrhea [36].
	Caffeic acid			
16.	Rubiadin	*Rubia cordifolia* L. (Rubiaceae)	Mishtu	Immune-related diseases inflammation, urinary infections, bone ache, skin diseases, vertigo, insomnia, rheumatism, tuberculosis, hematemesis, menstrual disorders, contusions [37].
	Mollugin			
17.	Verbascoside	*Verbascum thapsus* L. (Scrophulariaceae)	Janglitamaku	Pain, muscle spasm, bleeding, nerve tonic, wounds, allergy, cancer, oxidative stress, blood pressure, anxiety, inflammation, sepsis, diuretic, cough, skin diseases, cuts, wounds and swelling, diarrhea [38,39].
	Aucubin			
18.	Solasonine	*Solanum americanum* Mill. (Solanaceae)	Bara lianchu	Healing, dental caries, bladder spasm, joint pains, cooling, cough, gastric ulcer, protozoal infections, diabetes, inflammation [40,41].
	Solamargine			
19.	Pennogenin	*Trilliumgovanianum* Wall. Ex D. Don (Melanthiaceae)	Nag Chhatri	Dysentery, wounds, inflammation, antiseptic, boils, menstrual and sexual disorders, pain, inflammation, Leishmanial infection, cancer, wound [42,43].
	2,4-Decadienal			
20.	Protocatechuic acid	*Valeriana jatamansi* Jones (Caprifoliaceae)	Nihani	Cuts, wounds, skin disorders, analgesic, anxiety disorder, tranquilizing hypnotic, irritable bowel syndrome, epilepsy, snake poisoning, hyperlipidemia, depressive insomnia, rotavirus enteritis [44,45].
	Valtrate			
21.	Methyl salicylate	*Viola canescens* Wall. (Violaceae)	Banksha	Cough, cold, fever, jaundice, malaria, protozoa infection, cancer, flatulence, inflammation or irritation, bleeding abrations, fever, respiratory problems, sepsis, fever [46,47].
	Emetine			
22.	β-sitosterol	*Girardinia diversifolia* (Link) Friis (Urticaceae)	Zaran	Cytotoxic, Snake bite, Muscles sprain, constipation, headaches, fever, ringworm, gastric troubles, eczema, chest and joint pain, rheumatism, tuberculosis, headache, joint aches, diabetes, asthma, stomach inflammation, gonorrhea, delivery problems, bone fracture, internal injury, blood purification [48,49,50].
	Scopoletin			
23.	Atropine	*Datura stramonium* L. (Solanaceae)	Dhatura	Asthma, inflammation, pain and spasm in irritable bowel, gout, madness, epilepsy, depression, burns, rheumatism Parkinson’s disease, piles, pain [51].
	Scopolamine			
24.	Eugenol	*Ocimum sanctum* L. (Labiatae)	Tulsi	Bronchial asthma, fever, cold, cough, malaria, dysentery, convulsions, diarrhea, arthritis, skin diseases, insect bites, gastric, liver and heart disorder, diabetes stomachache, headache, inflammation, tuberculosis, stress, poisoning, leukoderma [52].
	Cirsilineol			
25.	Charantin	*Momordica charantia* L. (Cucurbitaceae)	Bitter Gourd	Cholesterol, HIV, gout, jaundice, abdominal pain, kidney (stone), rheumatism, fever, scabies, ulcer, inflammation, leukemia, diabetes, tumor, diabetes [53,54].
	Momordicine			
26.	Gingerol	*Zingiber officinale Roscoe* (Zingiberaceae)	Ginger	Inflammation, nausea, analgesic, fever, dysentery, heartburn, flatulence, diarrhea, diabetes, carminative, stimulant to GIT, relieve spasm of involuntary muscle, digestion, vasodilation, cough, asthma, pain, flatulence, constipation [55].
	Lariciresinol			
27.	Withanone	*Withania somnifera* (L.) Dunal (Solanaceae)	Ashwagandha	Abortion, clear or open the natural ducts of the fluids and secretions, pain, promoting calm and sleep, miscarriage, post-partum difficulties, inflammation, tumor, stress, oxidative stress, mind-booster, rejuvenation [56].
	Withaferin A			
28.	Geraniin	*Phyllanthus emblica* L. (Phyllanthaceae)	Indian gooseberry	Tumor, pain, fever, stress, inflammation, oxidative stress, depression, liver damage, ulcer, radioprotective, diabetes, cancer, wound healing, cytotoxic [57,58].
	Phyllanthin			
29.	Allicin	*Allium sativum* L. (Amaryllidaceae)	Garlic	Cold, influenza, dyspepsia, loss of appetite, snake bites, stress, inflammation, diabetes, aging effects, cancer, lung disorders, whooping cough, stomach disorders, cold, earache, cardiovascular disorder, Alzheimer’s disease [59].
	Pyrogallol			
30.	Quercitrin	*Bryophyllum pinnatum* (Lam.) Oken (Crassulaceae)	Pattharcaṭṭa	Ulcer, inflammation, analgesic, jaundice, kidney stones, respiratory tract infections, boils, insect bites, hypertension, diabetes, cancer, HPV [60].
	Bryophyllin A			
31.	Alpha-pinene	*Pinus roxburghii* Sarg. (Pinaceae)	Chir pine	Dyslipidemia, oxidative stress, wound healing, analgesic, inflammation, cytotoxic [61].
	Abietic acid			
32.	Thymoquinone	*Nigella sativa* L. (Ranunculaceae)	Black cumin	Asthma, hypertension, diabetes, inflammation, cough, bronchitis, headache, eczema, fever, dizziness, influenza, carminative, stimulant, diuretic [62].
	Thymol			
33.	Aloe-emodin	*Aloe barbadensis* Miller (Asphodelaceae)	*(Aloe vera* )	Burn injury, eczema, cosmetics, inflammation, fever, malaria [63].
	Emodin			
34.	Koenimbine	*Murraya koenigii* (L.) Spreng. (Rutaceae)	Curry tree	Piles, inflammation, itching, fresh cuts, dysentery, bruises, and edema, helminth infection, analgesics, digestives, and appetizers, oxidative stress, inflammation, nephroprotective [64,65].
	Mahanimbine			
35.	Asiaticoside	*Centella asiatica* (L.) Urb. (Apiaceae)	Brahma manduki	Ulcerous skin, weakness, burns, duodenal, stomach ulcers, lupus, antinociceptive, inflammation, scleroderma, leprosy vein disorder, neuroprotection, wound healing, eczema, dermatitis, psoriasis [66].
	Madecassoside			

**Table 2:** Table describing the active phytocompounds, plant source, binding energy, and interactive amino acids.

Name of Compound	Plant Source	Binding Energy (KJ/Mol)	No. of Hydrogen Bonds	Hydrogen Bonds	Interactive Amino Acids
Asiaticoside	*Centella asiatica* (L.) Urb.	−10.9	6	Ser137, Glu113, Leu115, Asp152, Ala110, Arg148	Ile106, Tyr59, Lys63, Asp124, Glu120, Ala149, Lys114, Ser112, Val111, Lys117, Cys134, Leu130, Arg136, Thr85, Ile88, Met70, Leu139, Phe155, Leu66, Tyr92, Leu156, Met140
Madecassoside	*Centella asiatica* (L.) Urb.	−9.7	5	Tyr59, Asp124, Ala110, Ala123, Cys67	Trp95, Leu66, Phe155, Thr85, Met70, Tyr92, Ser137, Leu139, Arg136, Leu130, Leu156, Ile88, Phe127, Lys63, Arg131, Pro128, Leu115, Asp152, Val11, Ala149, Glu120, Arg148, Met140, Ile106
Beta-sitosterol	*Girardinia diversifolia* (Link) Friis	−9.1	2	Thr85, Ser137	Tyr59, Ile106, Ala110, Asp152, Met140, Arg148, Leu139, Arg136, Val138, Ala81, Ile88, Leu156, Met70, Tyr92, Phe155, Leu66, Lys63
Bryophyllin A	*Bryophyllum pinnatum* (Lam.) Oken	−10.6	2	Thr85, Cys67	Phe155, Arg148, Met140, Cys134, Ile88, Leu139, Ser137, Arg136, Ala81, Met84, Lys63, Leu66, Met70, Ile106, Tyr59, Leu156, Ala110, Asp152
Mahanimbine	*Murraya koenigii* (L.) Spreng	−9.4	-	-	Arg136, Ser137, Thr85, Cys67, Tyr59, Ile106, Ala110, Leu156, Lys63, Tyr92, Leu66, Phe155, Met70, Ile88, Leu139, Leu130
Pennogenin	*Trilliumgovanianum* Wall. Ex D.Don	−10.3	1	Arg148	Cys134, Met84, Ile88, Met70, Tyr92, Arg107, Phe155, Ile106, Leu156, Ala110, Leu66, Lys63, Met140, Tyr59, Leu139, Ser137, Arg136, Thr85, Ala81
Rutin	*Berberis aristata DC.*	−9.2	3	Ser137, Thr85, Tyr59	Cys134, Met84, Glu120, Asp152, Arg148, Ala110, Leu156, Ala123, Asp124, Tyr92, Leu66, Phe155, Met70, Lys63, Cys67, Phe127, Arg136, Leu130, Ile88, Leu139
Solasonine	*Solanum americanum* Mill.	−9.7	2	Arg148, Ser137	Glu146, Asp145, Lys117, Glu120, Met70, Tyr92, Leu66, Phe155, Asp152, Met140, Arg136, Leu139, Leu130, Lys68, Ile88, Arg131, Asp124, Ala149
Solamargine	*Solanum americanum* Mill.	−9.5	5	Arg131, Lys63, Asp124, Asp145, Arg148	Phe127, Ala123, Leu130, Arg136, Glu120, Lys117, Tyr59, Leu139, Ser137, Met140, Thr85, Ile88
Withaferin A	*Withania somnifera* (L.) Dunal	−10.2	2	Tyr59, Tyr92	Ile106, Ala110, Leu66, Lys63, Phe155, Arg148, Leu139, Val138, Thr85, Trp185, Ser137, Met84, Cys134, Arg136, Ile88, Leu130, Met140, Leu156
Withanone	*Withania somnifera* (L.) Dunal	−9.3	-	-	Ile106, Leu66, Lys63, Arg148, Tyr92, Met70, Leu130, Arg136, Ile88, Cys134, Thr85, Met84, Ser137, Leu139, Met140, Phe155, Asp152, Ala110, Leu156, Tyr59
Chenodeoxycholic acid	Bile component	−10.8	4	Ser137, Thr85, Asp152, Tyr59	Val138, Cys134, Met84, Leu139, Ile88, Arg148, Met140, Phe155, Leu156, Ile106, Ala110, Lys63, Tyr92, Leu66, Met70, Arg136

**Table 3:** Drug likeness prediction of 11 active phytocompounds of 8 medicinal plants.

Phytocompounds	miLogP	TPSA	MW	nON	nOHNH	Nviolations
Chenodeoxycholic acid	4.25	77.75	392.28	4	3	0
Asiaticoside	0.37	315.21	959.13	19	12	3
Beta-sitosterol	8.62	20.23	414.72	1	1	1
Bryophyllin A	2.09	115.44	472.53	8	2	0
Madecassoside	−0.55	335.44	975.13	20	13	3
Mahanimbine	7.10	25.02	331.46	2	1	1
Pennogenin	4.99	58.92	430.63	4	2	0
Rutin	−1.06	269.43	610.52	16	10	3
Solasonine	1.40	258.72	884.07	17	10	3
Solamargine	2.41	238.49	868.07	16	9	3
Withaferin A	3.86	96.36	470.61	6	2	0
Withanone	4.15	96.36	470.61	6	2	0

miLogP—Molinspiration LogP (To measure lipophilicity), TPSA—topological polar surface area, MW—Molecular wait, nON—hydrogenbonds acceptor, nOHNH—hydrogen bonds donors, nviolations—Number of violations.

**Table 4:** Toxicity prediction of active phytocompounds and bile component.

Phytocompounds	ProTox-II					
	LD_50_(mg/kg)	Hepato-Toxicity	Carcino-Genecity	Immuno Toxicity	Muta-Genicity	Cyto-Toxicity
Chenodeoxycholic acid	2000 (Class 4)	Active	Inactive	Inactive	Inactive	Inactive
Asiaticoside	4000 (Class 5)	Inactive	Inactive	Active	Inactive	Inactive
Beta-sitosterol	890 (Class 4)	Inactive	Inactive	Active	Inactive	Inactive
Bryophyllin A	31 (Class 2)	Inactive	Inactive	Active	Inactive	Active
Madecassoside	1190 (Class 4)	Active	Inactive	Active	Inactive	Inactive
Mahanimbine	4000 (Class 5)	Inactive	Inactive	Active	Inactive	Inactive
Pennogenin	1190 (Class 4)	Active	Inactive	Active	Inactive	Inactive
Rutin	1190 (Class 4)	Active	Inactive	Active	Inactive	Active
Solasonine	500 (Class4)	Inactive	Inactive	Active	Inactive	Active
Solamargine	1190 (Class 4)	Inactive	Inactive	Active	Inactive	Active
Withaferin A	300 (Class 3)	Inactive	Inactive	Active	Inactive	Active
Withanone	7 (Class 2)	Inactive	Inactive	Active	Inactive	Active

**Table 5:** Thermodynamic binding energy of Beta-sitasterol in complex with 6IE9.

Title	MMGBSA (kcal/mol)
Frame 1	−105.8395994
Frame 2	−122.4871042
Frame 3	−131.2524436
Frame 4	−113.052146
Frame 5	−139.4301793
Frame 6	−154.7243297
Frame 7	−149.2144921
Frame 8	−157.1657804
Frame 9	−164.8064715
Frame 10	−148.5722473

## Data Availability

All data are included in this manuscript.
